# Validity and responsiveness of the EQ-5D in assessing and valuing health status in patients with somatoform disorders

**DOI:** 10.1186/1477-7525-11-3

**Published:** 2013-01-10

**Authors:** Christian Brettschneider, Hans-Helmut König, Wolfgang Herzog, Claudia Kaufmann, Rainer Schaefert, Alexander Konnopka

**Affiliations:** 1 Department of Medical Sociology and Health Economics, University Medical Center Hamburg-Eppendorf, Martinistraße 52, 20246, Hamburg, Germany; 2 Department of General Internal Medicine and Psychosomatics, University of Heidelberg, Im Neuenheimer Feld 410, 69120, Heidelberg, Germany

**Keywords:** EQ-5D, Quality of life, Somatoform disorders, Medically unexplained symptoms, Validity, Responsiveness

## Abstract

**Background:**

The EQ-5D is a generic questionnaire providing a preference-based index score applicable to cost-utility analysis. This is the first study to validate the EQ-5D in patients with somatoform disorders.

**Methods:**

Data of the EQ-5D descriptive system, the British and the German EQ-5D index and the EQ Visual Analogue Scale, the Patient Health Questionnaire 15, the Patient Health Questionnaire 9, the Whiteley Index 7 and the Short Form 36 were collected from 294 patients at baseline, 244 at 6 months and 256 at 12 months after baseline.

The discriminative ability of the EQ-5D was evaluated by comparison with a general population sample and by the ability to distinguish between different symptom severities. Convergent validity was analysed by assessing associations between the EQ-5D and the other instruments. Responsiveness was evaluated by analysing the effects on scores between two measurements in groups of patients reporting worse, same or better health. The Bonferroni correction was employed.

**Results:**

For all items of the EQ-5D except ‘self-care’, patients with somatoform disorders reported more problems than the general population. The EQ-5D showed discriminative ability in patients with different symptom severities. For nearly all reference instruments there were significant differences in mean scores between respondents with and without problems in the various EQ-5D items and strong correlations with the EQ Visual Analogue Scale and the EQ-5D index scores. Evidence for the responsiveness of the EQ-5D could only be found for patients with better health; effects were medium at the utmost.

**Conclusions:**

The EQ-5D showed a considerable validity and a limited responsiveness in patients with somatoform disorders.

**Trial registration:**

Current Controlled Trials ISRCTN55280791

## Background

According to the Food and Drug Administration (FDA) Guidance document concerning patient-reported outcomes, the application of an existing patient-reported instrument to a new population requires a revalidation of the instrument in question [1]. The EQ-5D is a preference-based, generic index instrument measuring health related quality of life (HRQOL). Preference-based index scores are adopted in the calculation of quality adjusted life years (QALY) and hence possess pivotal relevance in the field of economic evaluation [2,3]. The EQ-5D is the most frequently used instrument in the calculation of QALY [4]. The psychometric properties of the EQ-5D have been demonstrated in populations with different diseases and disorders (e.g. inflammatory bowel disease [5], myocardial infarction [6], type-2 diabetes [7], schizophrenic, schizotypal and delusional disorders [8] burn injured adults [9], anxiety disorders [10]). However, in patients with somatoform disorders evidence of these properties is still missing. The diagnostic category “somatoform disorders” is used in both, ICD-10 [11] and DSM-IV [12] classification systems. It includes several disorders where a high number of medically unexplained symptoms is the main feature, for which adequate somatic examination does not reveal sufficient explanatory pathology. Referring to the prevalence rate, somatoform disorders are common. It can be assumed that 15% to 20% of patients in primary care suffer from a somatoform disorder [13-15].

The purpose of this study was to analyse the psychometric properties of the EQ-5D in patients with somatoform disorders. More precisely, we focused on discriminative ability (Does the instrument discriminate between different states of the disorder?), construct validity (Does the instrument measure the underlying construct to an appropriate extent?) in terms of convergent validity (Are the instrument scores correlated to the scores of instruments theoretically related?) and responsiveness of the EQ-5D (Does the instrument detect health state changes that occur over time?).

## Methods

### Study design

The origin of the data in this study is a cluster randomised controlled trial (CRCT) designed to evaluate a 3-month disorder-specific group intervention for patients with somatoform disorders conducted by their general practitioner (GP) and a psychosomatic specialist together in the GP’s office. It is called the “speciAL” trial (specific collaborative group intervention for patients with somatoform disorders in generAL practice) (ISRCTN55280791) [16].

### Patient recruitment

The study sample was recruited by 35 GPs located in the Rhine-Neckar area of south Germany. Inclusion criteria were: (1) persistent (≥6 months) bodily complaints without sufficient somatic explanation after systematic differential diagnostic work-up according to the assessment of the specifically trained GP; (2) medically unexplained symptoms (MUS) as the main treatment issue. Exclusion criteria were: age below 18 or above 70 years, residing further than 20 miles away from the respective practice; ongoing psychotherapy; substance abuse; severe psychiatric disorder (major depression, psychosis, dementia, etc.); severe organic disease (operationalized by Karnofsky index <70% [17]); being unable to complete the questionnaire; ongoing medico-legal proceedings due to disability pension or compensation for personal suffering. After consenting, patients were given a screening questionnaire to determine study eligibility. Therefore, the GPs’ patient recruitment had to be validated by a positive score on at least one of two somatization screeners: (1) At least mild somatic symptom severity on the Patient Health Questionnaire (PHQ-15) represented by a cutpoint of 5 [18] and/ or (2) relevant health anxiety on the Whiteley-7 (WI-7) [19]. Consistent with Christensen et al. [20], the 5-point Likert scale of the WI-7 was dichotomized (0=not at all/ a little, 1=moderately/ quite a bit/ extremely) for screening purposes. A sum score of ≥4 was used as indicative of relevant health anxiety [21,22]. Eligible patients were sent the complete baseline questionnaire. The recruitment of patients was conducted between November 2007 and December 2009. 304 patients were included in the analysis of the CRCT. Data were collected at baseline (t0), six months after baseline (3 months after intervention) (t1) and 12 months after baseline (9 months after intervention) (t2). Design and results of this trial have been reported elsewhere [23]. The analysis presented in this article is based on a sample of 294 patients. The difference follows from EQ-5D questionnaires not returned from patients. Since this study is a validation study, it is neither reasonable nor necessary to separate the study sample into an intervention and a control group nor to evaluate the groups for imbalances.

### Measures

*EQ-5D:* The EQ-5D is composed of five items concerning `mobility´ (problems in walking about), `self-care´ (problems with washing or dressing), `usual activities´ (problems with performing usual activities – e.g. work, study, housework, family or leisure activities), `pain/discomfort´ and `anxiety/depression´ [24]. The response options are located on a three level ordinal scale describing `1 – no problems´, `2 – moderate problems´ and `3 – severe problems´. This layout is called the *EQ-5D descriptive system*. The result of the descriptive system is called the *EQ-5D self-reported health state*, a five-digit code specifying a specific health state (e.g. 11223 = no problems in `mobility´, no problems in `self-care´, moderate problems in `usual activities´, moderate problems in `pain/ discomfort´, severe problems in `anxiety/ depression´). Theoretically, 243 (3^5^) different health states can be defined by the EQ-5D descriptive system. It is possible to assign an index score of quality of life to each self-reported health state. The mentioned index scores are based on a survey of the general population in which participants were ask to assign utilities to different self-reported health states. Thus the EQ-5D index represents the valuation of the patient’s health state from a general population’s perspective (full health = 1.0). The index scores employed in this study are based on a British tariff developed by Dolan et al. (EQ-5D index UK) [25] and -alternatively- a German tariff developed by Greiner et al. (EQ-5D index D) [26]. In addition to the descriptive system, the EQ-5D includes a visual analogue scale (*EQ VAS*). The EQ VAS is a rating scale ranging from 0 (worst imaginable health state) to 100 (best imaginable health state) and represents the valuation of the health state from the patient’s point of view. The respondent is asked to mark his actual health state on this scale. The validated German version of the EQ-5D was used in this study [26].

#### Patient Health Questionnaire 15 (PHQ-15)

The PHQ-15 is a subscale of the full PHQ and is applied for the assessment of somatic symptom severity [27,28]. In patients with somatoform disorders the questionnaire features a high internal reliability and convergent as well as divergent validity [28]. It consists of 15 items covering the most frequent symptoms in somatoform disorders due to DSM-IV [28].

The scores of all 15 items can be accumulated to an overall score between 0 and 30. A score between 0 and 4 represents a minimal somatic symptom severity, a score between 5 and 9 a low, between 10 and 14 a medium and scores greater than or equal to 15 a high somatic symptom severity [28]. The validated German version of the PHQ-15 was used in the present study [29].

#### Patient Health Questionnaire 9 (PHQ-9)

The PHQ-9 is a subscale of the full PHQ and is applied for the assessment of depression severity [30]. The PHQ-9 has not been validated in a population of patients suffering from somatoform disorders. However, because of its documented general usability in the assessment of depression, we employed this questionnaire [30]. It contains 9 items, checking the DSM-IV symptoms of depression. The overall score of the PHQ-9 ranges from 0 to 27. Depression severity is classified as minimal (score 0 to 4), mild (5 to 9), moderate (10 to 14), moderately severe (15 to 19) or severe (20 to 27) [30]. The validated German version of the PHQ-9 was used in the present study [31].

#### Whiteley Index 7 (WI-7)

The WI-7 is a screening instrument for illness worries representing a cognitive/ emotional approach [19]. The WI-7 was derived from the original Whiteley-Index [19,32] and has acceptable psychometric properties in patients with somatoform disorders [19]. It contains seven questions relating to worries about health, body, symptoms, illness or accuracy of diagnosis. The response options for the questions are displayed on a 5-point Likert scale. The WI-7 overall score ranges from 0 to 28. Higher scores are indicating more severe disease states. The validated German version of the WI-7 was used in this study [33,34].

#### Short Form 36 (SF-36)

The SF-36 is a health related quality of life questionnaire consisting of 36 items which compose 8 health concepts forming a health profile [35,36]. These health concepts are physical functioning (PF), physical role functioning (RP), bodily pain (BP) and general health perceptions (GH) as well as vitality (VT), social functioning (SF), emotional role functioning (RE) and mental health (MH). The dimensional scores reach values from 0 (worst health state) to 100 (best health state). Furthermore, two component scores – physical (PCS) and mental (MCS) – can be calculated by summarizing the weighted dimensional scores. The German standard version of the SF-36 was used in the present study [37]. Although not formally validated in patients with somatoform disorders, the SF-36 has been frequently used in this population [38-41].

### Psychometric analyses

*Discriminative ability* refers to the ability of a measure to distinguish between different health states [42]. We hypothesised that the EQ-5D is able to distinguish between patients with somatoform disorders and the general population and to differentiate between patients with different severities of illness. For the comparison with the general population data from a representative survey (n = 3552) using the EQ-5D in the German general population in 2002/2003 were available [43].

To evaluate the discriminative ability between patients with different severities of disease the relationship of the EQ-5D descriptive system, the EQ VAS and the EQ-5D index scores to the somatic symptom severity was analysed. We chose the PHQ-15 as it depicts the severity of somatoform disorders. To assess the EQ VAS score and the score of the British and German EQ-5D index for ceiling effects, the distribution of these scores was analysed by percentiles and by the proportion of patients achieving the highest possible scores.

*Construct validity* applies to the ability of an instrument to reproduce the underlying construct in a reasonable manner [44]. We analysed only a part of construct validity, namely convergent validity. This means we focussed on the correlation of the EQ-5D with instruments which are based on related theoretical constructs [42]. We hypothesised that there is an association between the EQ-5D and measurements of psychopathology, symptoms and quality of life on the item level, the level of the EQ VAS and the level of the EQ-5D index scores. The reference instruments in the evaluation of the EQ-5D were the PHQ-15, PHQ-9, WI-7 and the SF-36.

*Responsiveness* refers to the capability of an instrument to detect changes of the health state over time [45]. We hypothesised that the EQ-5D is responsive. We used the disease specific PHQ-15 to analyse the responsiveness of the EQ-5D and furthermore employed the health transition question of the generic SF-36 as a measure of change to perform the analysis from a generic perspective as well. The health transition question does not contribute to any of the eight health concepts or of the two component scores of the SF-36 [46]. The transition question is a 5-point Likert scale ranging from `much´ and `somewhat better´ over `about the same´ to `somewhat´ and `much worse´, which reports the actual health state in comparison to the health state one year ago. In line with this analysis, the responses “much” or “somewhat better” and “much” or “somewhat worse” are summarised as “better” and “worse” health respectively. “About the same” is labelled as “same” or “unchanged” health.

### Statistical methods

Concerning the statistical methods, two facts have to be highlighted. First, the categories `moderate problems´ and `severe problems´ of the EQ-5D descriptive system were pooled into one category `problems´, as `severe problems´ occurred rarely. Therefore, for all analyses of convergent validity on item level, the items of the EQ-5D descriptive system were used as dichotomous variables (no problems; problems). Second, as the EQ VAS and both EQ-5D index scores showed no normal distribution, non-parametric methods were employed. No adjustment for clustering of patients around GPs was performed, as all observed ICCs for baseline values were smaller than 0.1, indicating a low level of relatedness of patient characteristics within the clusters [23].

To assess *discriminative ability*, the χ^2^-test (EQ-5D items) and the Kruskal-Wallis test (EQ VAS and EQ-5D index scores) were used. The analysis of the discriminative ability was based on the baseline data.

Because of the different nature of the data of the EQ-5D descriptive system on the one hand and of the EQ-5D index scores and the EQ VAS on the other hand we had to choose different statistical approaches in the evaluation of *convergent validity*. The Mann–Whitney test was applied and the effect size (Cohen’s d) was calculated for assessing the EQ-5D descriptive system. The Spearman rank correlation coefficient (r_s_) was calculated for assessing the EQ VAS and the EQ-5D index scores. According to Cohen, a correlation was considered small for 0.1 ≤ |r_s_| < 0.3, moderate for 0.3 ≤ |r_s_| < 0.5 and large for |r_s_| ≥ 0.5 [47]. The analysis of the convergent validity was based on the baseline data. In order to identify not only the hypothesized correlations and effects but also those unexpected, we performed a complete analysis for the items of the EQ-5D, the EQ-5D index scores and the EQ VAS.

*Responsiveness* was evaluated measuring the mean differences, effect sizes (ES) and standardised response means (SRM) in the groups of patients reporting worse, same and better health on the SF-36 transition question, and by the Spearman rank correlation coefficient (r_s_) of the changes scores of the PHQ-15 and the EQ-5D. ES was calculated as follows: ES = (M_x_ – M_0_)/ SD_Baseline_. M_0_ denotes the mean score of the baseline assessment, M_x_ the mean score of the follow-up assessment at time x. SD_Baseline_ is the standard deviation of the baseline assessment. SRM was defined as: SRM = (M_x_ – M_0_) / SD_MX – M0_. The numerator is the same as in case of the ES, the denominator is the standard deviation of the difference in scores. Scores of ES and SRM ≥ |0.8| were considered as large effect, scores from ≥ |0.5| to < |0.8| as medium, scores from ≥ |0.2| to < |0.5| as small and scores ≥ |0.1| to |0.2| as trivial [47]. As we employed the transition question of the SF-36 which covers the change in health compared to one year before, the evaluation of responsiveness focussed on the period between t0 and t2.

For statistical testing, the level of significance was defined at α = 0.05. Since several hypotheses are tested per item and score of the EQ-5D, the Bonferroni correction was performed. As 13 different scores were used for assessing the convergent validity of the EQ-5D, the level of significance was defined at α = 0.05 / 13 = 0.0038. Analyses were performed using the Statistical Package for the Social Sciences (version 18.0, SPSS Inc., Chicago, IL, USA).

## Results

### Sociodemographic characteristics

The baseline sociodemographic characteristics of the 294 analysed patients are displayed in Table 1. The mean age of the analysed sample was 49.06 years (SD: 12.51). The majority of participants was female (74.9%), married or living with a partner (69.8%) and possessing a secondary school graduation (73.2%).

**Table 1:** Sociodemographic characteristics of the study sample (n = 294)

**Age in years**	
mean (SD)	49.06 (12.51)
median	49
Gender	
male	25.2%
female	74.8%
Martial status^a^	
unmarried	20.0%
married	65.6%
separated/ divorced	10.3%
widowed	4.1%
Living arrangement^b^	
living alone	22.3%
living with spouse/ partner	70.0%
living withe parents/ relatives	4.9%
other	2.8%
Education^c^	
no school graduation	1.4%
secondary school graduation	73.1%
technical college/ university entrance qualification	13.3%
college/ university degree	11.9%
other (e.g. school for mentally handicapped children)	0.3%

^a^ 4 missing values of this variable (n= 290).

^b^ 7 missing values of this variable (n = 287).

^c^ 8 missing values of this variable (n = 286).

### Descriptive statistics of the EQ-5D

86.7% of patients reported problems in the item `pain/ discomfort´, 66.3% in the item `anxiety/ depression´, followed by `usual activities´ (47.4%), `mobility´ (28.6%) and `self-care´ (3.1%). Only 6.8% of patients reported no problems at all (Table 2). The most frequently reported health state was 11122, which indicates moderate problems in the items `pain/ discomfort´ and `anxiety/ depression´. Health states with more than one scaling of `severe problems (3) were hardly reported. The distributions of the EQ VAS and the EQ-5D index scores are displayed in Table 3. The mean EQ VAS score was 58.56 (SD: 19.99) (0 to 100 = worst to best imaginable health state). The mean scores of the EQ-5D index UK and the EQ-5D index D were 0.62 (SD: 0.27) and 0.77 (SD: 0.24) respectively (full health = 1.0).

**Table 2:** EQ-5D descriptive system: most frequently reported EQ-5D self-classified health states

**EQ-5D health state (regarding five items in the following order: `mobility´, `self-care´, `usual activities´, `pain/ discomfort´,`anxiety/ depression´**	**n (%)**
11122^*^	57 (19.5%)
11222	42 (14.2%)
11121	41 (13.9%)
21222	25 (8.5%)
11111	20 (6.8%)
11112	16 (5.4%)
21221	13 (4.4%)
11221	9 (3.1%)
21232	8 (2.7%)
11223	7 (2.4%)

This table lists all health states which were reported at least five times among all respondents. Hence percentages do not sum up to 100%. There was one missing value for the EQ-5D self-classified health state (n=294). ^*^1 – no problems, 2 – moderate problems, 3 – severe problems.

**Table 3:** Scores of instruments

**Measures**		**Range: worst - best**	**n**	**Score**					
				**Mean (SD)**	**Median (range)**				
Health-related quality of life	EQ VAS	(0 – 100)	287	58.56 (19.99)	60 (0–100)
	EQ-5D index UK	(−0.594 – 1.00)	293	0.62 (0.27)	0.73 (−0.18 – 1.00)
	EQ-5D index D	(−0.2 – 1.00)	293	0.77 (0.24)	0.89 (0.11 – 1.00)
	SF-36				
	Physical component score (PCS)	(2–76)	294	42.78 (9.11)	42.96 (13.99-65.40)
	Mental component score (MCS)	(1–81)	294	40.86 (10.61)	40.61 (14.15-64.66)
	Physical functioning (PF)	(0–100)	294	70.86 (24.10)	75 (0–100)
	Role physical (RP)	(0–100)	294	68.62 (26.36)	75 (0–100)
	Bodily pain (BP)	(0–100)	294	48.50 (25.54)	41 (0–100)
	General health (GH)	(0–100)	294	49.75 (18.54)	50 (5–100)
	Vitality (VT)	(0–100)	294	39.75 (20.33)	40 (0–100)
	Social functioning (SF)	(0–100)	294	61.97 (28.08)	62.50 (0–100)
	Role emotional (RE)	(0–100)	294	67.58 (27.22)	66.67 (0–100)
	Mental health (MH)	(0–100)	294	54.71 (19.68)	56 (8–100)
Symptoms	PHQ-15 Sum score	(30–1)	294	12.66 (4.81)	12 (2–26)
	PHQ-9 Sum score	(27–0)	294	9.33 (5.35)	9 (0–27)
Illness worries	WI-7 Sum score	(28–0)	294	10.91 (6.54)	10 (0–28)

### Scores of reference instruments

The scores of the reference instruments are presented in Table 3. The mean score of the *PHQ-15* was 12.66 (SD: 4.81) representing a medium severity of somatoform disorder. 84 patients (29%) suffered from a minimal or low (PHQ-15: 0–9), 110 patients (37%) from a medium (PHQ-15: 10–14) and 100 patients (34%) from a high somatic symptom severity (PHQ-15: 15–30). The *PHQ-9* showed a mean score of 9.33 (SD: 5.35) describing mild to moderate severity of depression. The WI-7 displayed a mean of 10.91 (SD: 6.54). The mean scores for the SF-36 health concepts PF, RP, SF and RE were located between 60 and 70, for the health concepts BP, GH and MH around 50 and the score for VT nearly 40. The values of the PCS and the MCS were around 40.

### Discriminative ability

**Figure 1:**
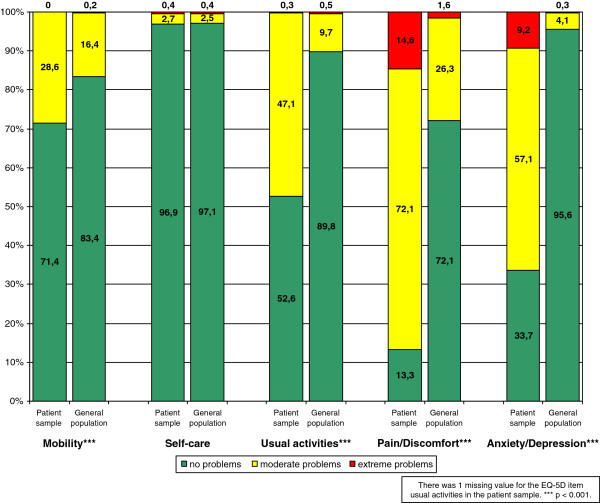
**Distribution of responses to items of EQ-5D descriptive system in patient sample (n = 295) and general population (n = 3552)**[43]**.**

Figure 1 shows the discriminative ability of the EQ-5D in terms of the differences between the study population and a general population sample from Germany (n = 3552) [43]. The study population of patients with somatoform disorders reported significantly more problems in four of five items of the EQ-5D (p < 0.001). Only in the item `self-care´, there was no difference apparent.

The maximum score of the EQ VAS, the EQ-5D index UK and the EQ-5D index D was reached by only 1%, 6.8% and 12.6% of patients with somatoform disorders (data not shown). Considering the percentiles of the distribution of the EQ-5D scores, there was no distinct ceiling effect for the EQ VAS and the EQ-5D index UK but for the EQ-5D index D (data not shown).

The ability of the EQ-5D to discriminate between different somatic symptom severities is presented in Table 4. In every item of the EQ-5D descriptive system the proportion of patients with problems in the particular grade of somatic symptom severity increased with the increase of somatic symptom severity. Furthermore, the valuation of the health state decreased with the increase of somatic symptom severity. For the EQ-5D descriptive system, the EQ VAS and the EQ-5D index scores the differences in proportions and scores were significant.

**Table 4:** Association between somatic symptom severity (PHQ-15) and EQ-5D

	**Number of patients with problems in EQ-5D item:**						**EQ VAS Score**								**EQ-5D Index UK**										**EQ-5D Index D**											
**Somatic symptom severity(PHQ-15)**	**Mobility (%) **^**a**^	**Self-care (%) **^**a**^	**Usual Activity (%) **^**a**^	**Pain/ Discomfort (%) **^**a**^	**Anxiety/ Depression (%) **^**a**^	**Mean (SD)**	**Median**	**Mean (SD)**	**Median**	**Mean (SD)**	**Median**
● Minimal/ Low	14 (16.6%)	0 (0.00%)	22 (26.2%)	65 (77.4%)	44 (52.4%)	68.59 (18.75)	70	0.75 (0.16)	0.727	0.87 (0.14)	0.888
(0–9)											
(n=84 / 29%)											
● Medium	32 (29.1%)	2 (1.80%)	54 (51.8%)	96 (87.3%)	72 (65.5%)	58.8 (18.54)	60	0.65 (0.24)	0.691	0.80 (0.2)	0.888
(10–14)											
(n=110 / 37%)											
● High	38 (38.0%)	7 (7.00%)	63 (63.0%)	94 (94.0%)	79 (79.0%)	50.01 (18.72)	50	0.49 (0.32)	0.689	0.66 (0.3)	0.838
(15–30)											
(n=100 / 34%)											
p-value	0.006^b^	0.009^b^	< 0.001^b^	0.004^b^	0.001^b^	< 0.001^c^								< 0.001^c^										< 0.001^c^											

^a^ Proportion of patients with problems according to the EQ-5D item in the group of patients with low / medium / high disease severity ^b^ Chi-square test; ^c^ Kruskal-Wallis test; This analysis is based on baseline data.

### Convergent validity

Table 5 shows the associations between the response level of the EQ-5D items and the scores of the reference instruments. Displayed are the mean scores of the reference instruments categorized by the presentation of problems according to the EQ-5D items. Furthermore, effect sizes (Cohen’s d) are reported.

**Table 5:** Association between response level of EQ-5D items and score of other measures

**Other measures**		**Mean score of other measures by response level of EQ-5D item**																
	**EQ-5D item**	**Mobility**					**Self Care**								**Usual Activity**											**Pain/ Discomfort**														**Anxiety/ Depression**																
	**Problems:**	**No**	**Yes**	***d***^**a**^	**No**	**Yes**	***d***^**a**^	**No**	**Yes**	***d***^**a**^	**No**	**Yes**	***d***^**a**^	**No**	**Yes**	***d***^**a**^																																								
Symptoms	PHQ-15																
	● Sum score	11.99	14.4	−0.51^**^	12.54	17.00	−1.13^*^	11.3	14.23	−0.64^**^	9.56	13.15	−0.78^**^	11.17	13.44	−0.20^**^	
	PHQ-9																
	● Sum score	8.68	10.95	−0.43^**^	9.21	12.89	−0.61	7.57	11.29	−0.73^**^	7.18	9.65	−0.49	6.37	10.83	−0.94^**^	
Illness worries	WI-7																
	● Sum score	9.93	13.27	−0.52^**^	10.68	17.44	**−1.14**	8.83	13.08	−0.69^**^	7.92	11.34	−0.55^*^	8.34	12.17	−0.64^**^	
Health-related quality of life	SF-36																
	● Physical component score	45.76	34.18	1.61^**^	42.85	29.89	1.92^**^	46.44	38.13	1.04^**^	52.15	40.96	1.50^**^	43.05	42.14	0.10	
	● Mental component score	41.20	40.56	0.06	41.14	37.37	0.32	44.81	36.72	0.81^**^	43.81	40.59	0.30	49.00	36.97	1.32^**^	
	Physical functioning	80.14	46.84	1.70^**^	71.71	36.36	1.69^**^	81.22	59.25	1.03^**^	90.58	67.57	1.23^**^	75.02	68.39	0.28	
	Role physical	71.79	56.55	0.55^**^	68.51	33.33	1.23^**^	77.60	55.94	0.86^**^	84.62	64.80	0.87^**^	75.76	63.21	0.49^**^	
	Bodily pain	54.66	29.52	1.24^**^	48.32	20.78	1.46^**^	57.53	36.53	0.94^**^	76.92	42.98	1.45^**^	52.38	44.99	0.30	
	General health perceptions	53.06	41.10	0.72^**^	50.06	36.33	0.77	56.85	41.83	0.90^**^	63.92	47.45	0.95^**^	55.71	46.56	0.51^**^	
	Vitality	43.14	30.40	0.67^**^	39.97	24.63	0.75	48.94	28.85	1.17^**^	53.12	37.42	0.78^**^	50-57	33.88	0.90^**^	
	Social functioning	65.95	52.23	0.50^**^	62.50	47.22	0.53	75.57	47.93	1.17^**^	74.68	60.10	0.53^*^	78.41	53.72	0.97^**^	
	Role emotional	69.84	61.51	0.29	68.19	44.44	0.73	76.19	57.55	0.70^**^	79.49	65.62	0.53^*^	83.84	59.15	1.01^**^	
	Mental health	56.50	50.94	0.27	55.27	43.56	0.55	61.82	47.22	0.79^**^	62.77	53.71	0.48	68.29	48.12	1.17^**^	

There were missing values for some of the other measures used for comparison; the number of observations is presented in Table 3; p-values for differences in scores: ^*^ p < 0.003; ^**^ p < 0.001; ^a^ |*d*| = effect size (Cohen´s *d*), large effect sizes (|*d*| ≥ 0.8) are printed bold. This analysis is based on baseline data.

For nearly all reference instruments, significant differences in mean scores between respondents with and without problems in the various EQ-5D items were found.

The following results should be emphasised (p < 0.001) as they indicate convergent validity:

(1) Patients with moderate or severe problems in `mobility´ reported worse HRQOL in the SF-36 health concept `physical functioning´ than patients without problems. This difference represented an effect size of 1.7.

(2) Patients with moderate or severe problems in `pain/ discomfort´ reported worse HRQOL in the SF-36 health concept `bodily pain´ than patients without problems. This difference represented an effect size of 1.50.

(3) Patients with problems in `anxiety/ depression´ reported worse HRQOL in the SF-36 MCS and its health concepts (VT, SF, RE, MH) as well as worse scores in the PHQ-9 than patients without problems. All differences represented large effect sizes.

(4) Patients with problems in `mobility´, `self-care´, `usual activity´ and `pain/ discomfort´ reported worse HRQOL in the SF-36 PCS and most of its health concepts (PF, RP, BP, GH) than patients without problems. All differences represented large effect sizes.

The PHQ-15 and the WI-7, measuring somatoform complaints from a symptom perspective and with a cognitive/ emotional approach, respectively, showed significant differences, but only moderate effect sizes. The only exception was the item `self-care´. For this item large effect sizes were observed.

The evaluation of the EQ VAS and the EQ-5D index scores lead to similar results (Table 6): All correlations with the other measures were highly significant; most correlations between the reference instruments and the EQ VAS and the EQ-5D index UK were large. Exceptions were the PHQ-15, PHQ-9 and WI-7, showing only moderate correlations with the EQ VAS and the EQ-5D index scores, as well as the SF-36 MCS and the health concepts `mental health´, `role physical´ and `role emotional´. The EQ-5D index D showed moderate correlations mostly. Strong correlations were found with the PCS and the concepts `physical functioning´ and `bodily pain´ of the SF-36. The correlations between the EQ-5D index D on the one hand and the MCS and the concept `role emotional´ of the SF-36 on the other hand were only small.

**Table 6:** Correlation between EQ VAS score, EQ-5D index and scores of other measures

**Other measures**		**Correlation EQ VAS score**	**Correlation EQ-5D index UK**	**Correlation EQ-5D index D**
Symptoms	PHQ-15				
	● Sum score	−0.468	−0.448^**^	−0.392^**^	
	PHQ-9				
	● Sum score	−0.489	−0.474^**^	−0.345^**^	
Illness worries	WI-7				
	● Sum score	−0.471	−0.431^**^	−0.331^**^	
Health-related quality of life	SF-36				
	● Physical component score	**0.589**	0.574^**^	0.636^**^	
	● Mental component score	0.470	0.441^**^	0.221^**^	
	Physical functioning	**0.538**	0.567^**^	0.600^**^	
	Role physical	0.423	0.467^**^	0.386^**^	
	Bodily pain	**0.599**	0.579^**^	0.595^**^	
	General health perceptions	**0.633**	0.509^**^	0.464^**^	
	Vitality	**0.586**	0.555^**^	0.412^**^	
	Social functioning	**0.564**	0.530^**^	0.381^**^	
	Role emotional	0.382	0.436^**^	0.254^**^	
	Mental health	**0.517**	0.477^**^	0.308^**^	

Large correlations (|r_s_| ≥ 0.5) are printed bold; ^*^ p < 0.0038; ^**^ p < 0.001; the number of observations used for the calculation was 286 ≤ n ≤ 293, subject to missing values; the correlation of the EQ VAS score with the EQ-5D index UK was |r_s_| = 0.669 (p < 0.001); the correlation of the EQ-VAS with the EQ-5D index-D was |r_s_| = 0.635 (p < 0.001); the correlation of the EQ-5D index UK with the EQ-5D index D was 0.886 (p < 0.001). This analysis is based on baseline data.

### Responsiveness

Table 7 shows the responsiveness statistics. Anchored by the SF-36 transition question the EQ VAS and the EQ-5D index scores showed some effects after one year (between t0 and t2), especially in the group of patients reporting improved health. In this group the effect on the EQ VAS was medium, while the effect on the EQ-5D index scores was small. As expected there were only trivial effects in the group of patients reporting unchanged health. In the group of patients reporting worse health the effect on the VAS was trivial, while the effect on the EQ-5D index scores was small. The analysis by means of the disease specific PHQ-15 showed similar results (data not shown). We found highly significant, but only moderate correlations between changes in the PHQ-15 score and changes in the EQ VAS score (r_s_ = −0.311, p < 0.000) and significant small correlations between changes in the PHQ-15 score and changes in the EQ-5D index UK (r_s_ = −0.167, p = 0.011). The small correlation of the PHQ-15 and the EQ-5D index D (r_s_ = −0.144, ns) was not significant.

**Table 7:** Responsiveness of EQ VAS, EQ-5D index UK and EQ-5D index D based on SF-36 transition question as an external anchor of change

	**Statistics**	**Score**	**Change of health state anchored by SF-36 transition question**					
			**Worse (n=64)**	**Same (n=72)**	**Better (n=91)**			
t0 – t2	Mean difference (SD)	EQ VAS	−3.386 (19.714)	−3.284 (17.420)	10.744** (16.755)
		EQ-5D index UK	−0.065 (0.285)	0.029 (0.249)	0.076** (0.199)
		EQ-5D index D	−0.069 (0.266)	0.018 (0.229)	0.046* (0.181)
	Effect size	EQ VAS	−0.17	−0.14	0.62
		EQ-5D index UK	−0.18	0.11	0.39
		EQ-5D index D	−0.22	0.08	0.26
	Standardised response mean	EQ VAS	−0.16	−0.15	0.65
		EQ-5D index UK	−0.20	0.12	0.38
		EQ-5D index D	−0.23	0.07	0.25

Significant t-statistics (*p < 0.05; **p < 0.001) are printed bold.

## Discussion

To our knowledge, this study is the first to evaluate the psychometric properties of the EQ-5D in patients with somatoform disorders.

### Discriminative ability

The EQ-5D proved discriminative ability in somatoform disorders: It showed significant differences between patients with somatoform disorders and the general population. Large differences were especially found for the items `pain/ discomfort´ and `anxiety/ depression´. The only item showing no differences in comparison to the general population was `self-care´. For the EQ-5D self-reported health state no ceiling or floor effects were identifiable, as on the one side just 6.8% of patients reported a health state of 11111 without any problems, and on the other side health states with severe problems in more than one dimension were rarely reported. The same applies to the EQ VAS and the EQ-5D index UK. The EQ-5D index D showed a distinct ceiling effect. This effect results from the development of the German EQ-5D index. It was estimated based on a rather small general population sample of N = 334 and the 243 health states of the EQ-5D were derived from a set of 36 health states using a regression model. For this reason the EQ-5D index D has to be considered as less precise and preliminary. The consequence of this is that the health state 11112, i.e. no problems but moderate problems in the dimension anxiety / depression, is not connected with a loss of health related quality of life.

Furthermore, the results concerning the discriminative ability support the assumption that the EQ-5D items, the EQ VAS and the EQ-5D index scores are able to differentiate between patients with different severities of somatoform disorders.

### Convergent validity

Especially three items of the EQ-5D descriptive system were strongly associated with a generic reference instrument and thereby indicated convergent validity: Strong associations were found (1) between the EQ-5D item `anxiety/depression´ and the PHQ-9 as well as the SF-36/ MCS and its health concepts, (2) between the EQ-5D item `pain/discomfort´ and the SF-36 health concept `bodily pain´, and (3) between the EQ-5D item `mobility´ and the SF-36 health concept `physical functioning´. The other two EQ-5D items `usual activities´ and `self care´ definitely emphasise the physical health state, but also seem to be affected by the mental component [8,48]. Under this assumption the results confirm the presence of convergent validity for the EQ-5D item `usual activities´, as it showed associations with six of the eight SF-36 health concepts and with the PCS. Thus, physical and mental health concepts seem to be represented by this item. For the item `self-care´ the same cannot be stated, as it achieved large effect sizes only in physical health concepts and no significant results in the mental health concepts of the SF-36.

The associations between the EQ-5D items and the disorder-specific instruments were significant but only moderate in most instances. The evaluation of the EQ VAS and the EQ-5D index scores provided similar results. They correlated significantly with all dimensions of the generic and the specific instruments, but strong correlations, which were found mainly for the EQ VAS and the EQ-5D index UK, occurred primarily with the generic instruments.

This weak association with disorder-specific instruments deviates from the results of other studies which validated the EQ-5D in patients with mental disorders like anxiety disorder or schizophrenic, schizotypal and delusional disorders [8,10]. A possible explanation is that the PHQ-15 and the WI-7 are symptom measures, and their overall scores are symptom scores, whereas the EQ-5D scores (VAS, Index UK and Index D) represent a valuation of the health state. Additionally it has to be kept in mind that the PHQ-15 and the WI-7 are psychometric instruments describing a health state partially, whereas the EQ-5D scores are preference-based measures valuing the utility of a health state. As the reciprocity between psychometrically measured symptom status and preference-valued health status is not clear, it cannot be stated definitively whether and how the presence of individual symptoms is reflected in the valuation of a specific health state. It can be hypothesised that both constructs are not connected closely enough to show large correlations in this analysis.

Finally, it is worth mentioning, that there is no strong correlation between the EQ VAS or the EQ-5D index scores and the MCS. This could be explained by the nature of the EQ-5D, which consists of four items (`pain/ discomfort´, `mobility´, `self-care´, `usual activities´) that emphasise physical health primarily, and only one item (`anxiety/ depression´) focusing on mental health. Nevertheless, this is a problem that reduces the convergent validity of the EQ VAS and the EQ-5D index scores by a certain degree, as somatoform disorders are psychosomatic disorders after all, even though they become manifest in somatic symptoms.

### Responsiveness

For the assessment of responsiveness the use of the disease specific PHQ-15 as an anchor of change would be the method of choice. As there is no minimal important difference reported in the literature we chose the transition question of the SF-36 as an anchor. However, transition questions are considered as being biased [49]. For this reason we additionally employed a correlation analysis based on the PHQ-15 to support our findings. With respect to the responsiveness, it can be stated that the EQ-5D is responsive to a limited degree. In the group of patients with improved health on the transition question of the SF-36, the EQ VAS score and the EQ-5D index scores were responsive between t0 und t2. In the group of patients without changes in health, the EQ VAS score and the EQ-5D index scores showed no effect, which is desirable. In patients reporting worse health neither the EQ VAS nor the EQ-5D index scores was responsive. From the disease specific perspective of the analysis only the EQ VAS showed a moderate correlation to changes measured by the PHQ-15.

### Limitations

The main limitation of this study results from the study population. Only patients with persistent medically unexplained symptoms (MUS) were included in the study. This could limit the generalisability of the results. However, irrespective of the chronic courses of illness, the distribution of somatic symptom severity in the study population was well-balanced (Table 4). So even if an influence of the selected patient sample on the generalisability of the study has to be supposed, the principle of this study is not affected.

## Conclusions

The EQ-5D discriminated between patients with somatoform disorders and the general population, as well as between different severity states of somatoform disorders. The convergent validity of the EQ-5D items, the EQ VAS and the EQ-5D index scores was demonstrated. The EQ-5D items as well as the EQ VAS and the EQ-5D index UK show considerable associations with other measures of the constructs in question. The convergent validity of the EQ-5D index D is less pronounced yet present. The responsiveness of the EQ-5D index scores was limited; it was only significant for patients reporting an improved health state. In summary, the EQ-5D possesses a considerable validity and a limited responsiveness in patients with somatoform disorders.

## Abbreviations

BP: Bodily Pain; CRCT: Cluster Randomised Controlled Trial; EQ-5D: Euroqol-5D; EQ VAS: Euroqol-5D Visual Analogue Scale; ES: Effect Size; FDA: Food and Drug Administration; GH: General Health Perceptions; HRQOL: Health-related Quality of Life; ICD-10: International Classification of Diseases 10^th^ Revision; MCS: Mental Component Score of the SF-36; MH: Mental Health; MID: Minimal Important Difference; MUS: Medically unexplained symptoms; PCS: Physical Component Score of the SF-36; PF: Physical Functioning; PHQ: Patient Health Questionnaire; QALY: Quality Adjusted Life Year; RE: Role Emotional; RP: Role Physical; SF: Social Functioning; SF-36: Short Form-36; SRM: Standardised Response Mean; VT: Vitality; WHO: World Health Organization; WI-7: Whiteley Index-7.

## Competing interest

The authors declare that they have no competing interests.

## Authors´ contributions

CB, AK and HHK designed the study. WH and HHK obtained funding and supervised the study. CK and RS collected the data. CB analysed the data. All authors interpreted the data. CB drafted the manuscript. All authors critically revised the manuscript and approved the final version.

## Authors’ informations

Rainer Schaefert and Alexander Konnopka are both senior authors of this manuscript.
